# Robust undulatory locomotion through neuromechanical adjustments in a dissipative medium

**DOI:** 10.1098/rsif.2024.0688

**Published:** 2025-01-29

**Authors:** Kenta Ishimoto, Clément Moreau, Johann Herault

**Affiliations:** ^1^ Research Institute for Mathematical Sciences, Kyoto University, Kyoto 606-8502, Japan; ^2^ Nantes Université, École Centrale Nantes, IMT Atlantique, CNRS, LS2N, UMR 6004, Nantes F-44000, France

**Keywords:** animal locomotion, microswimmer, resistive force theory, coupled oscillators

## Abstract

Dissipative environments are ubiquitous in nature, from microscopic swimmers in low-Reynolds-number fluids to macroscopic animals in frictional media. In this study, we consider a mathematical model of a slender elastic locomotor with an internal rhythmic neural pattern generator to examine various undulatory locomotion such as *Caenorhabditis elegans* swimming and crawling behaviours. By using local mechanical load as mechanosensory feedback, we have found that undulatory locomotion robustly emerges in different rheological media. This progressive behaviour is then characterized as a global attractor through dynamical systems analysis with a Poincaré section. Furthermore, by controlling the mechanosensation, we were able to design the dynamical systems to manoeuvre with progressive, reverse and turning motions as well as apparently random, complex behaviours, reminiscent of those experimentally observed in *C. elegans*. The mechanisms found in this study, together with our dynamical systems methodology, are useful for deciphering complex animal adaptive behaviours and designing robots capable of locomotion in a wide range of dissipative environments.

## 1. Introduction

Biological gait patterns vary in different media, such as swimming in water with fins, gliding and flying in air with wings and walking and crawling on the ground with legs. Among this morphological diversity, undulatory locomotion for swimming and crawling is used by various species, from flagellated cellular locomotion in invertebrates to swimming and crawling by vertebrates, such as fish and snakes [1–6].

It has long been a major challenge to find the common biological mechanisms that enable adaptation to different mechanical environments. In some vertebrates, such as lamprey, central pattern generators (CPGs) in the nervous system have been well known as the key mechanism underlying gait control and environmental adaptation. CPGs are made of networks of neural oscillators in the spinal cord, where autonomous neuronal circuits produce cyclic motor activity. This rhythmic pattern generates basic locomotion and can be modulated by sensory feedback to adapt to the environment [7–11].

Animal mechanosensation has been intensively studied in fish undulatory swimming at moderate and large Reynolds numbers, with a focus on proprioceptive feedback, which uses perceptions of local body deformation to sustain a desired gait pattern through stretch feedback [12–14]. Similarly, these aquatic animals can also perceive the dynamics of the fluid, giving rise to exteroceptive sensory feedback, through their lateral line or mechanical receptors [15–17]. The role of exteroceptive feedback in local sensorimotor loops is an ongoing research subject, but recent experiments with fish-like swimming robots in water [9] found that a robust undulatory locomotion can be achieved by local exteroceptive feedback control. These vertebrates live in a large-Reynolds-number regime, where the inertia of the body and fluid plays a crucial role. However, undulatory locomotion is used even when the inertia is negligible, and this regime is the focus of this study.

**Figure 1.**
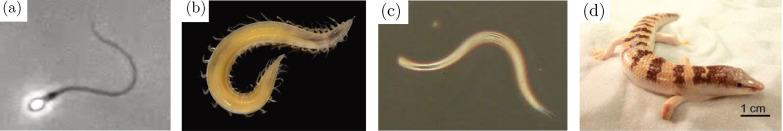
Examples of undulatory locomotion in a strongly dissipative medium. (*a*) Human sperm cells having whip-like motion without a neural network. Videomicroscopy from video 1 of the supplemental material of [18]. (*b*) Polychaeta worm, *Armandia brevis*, which can burrow in mud. Image adapted from [21] under CC BY-SA license (copyright 2024, Florida Museum of Natural History). (*c*) Swimming nematode captured and recorded by the authors at the north campus of Kyoto University. (*d*) Sandfish lizard that can swim in sand. Image adapted from [22] (copyright 2013, National Academy of Sciences).

Animal locomotion in dissipative media includes swimming in low-Reynolds-number flow and crawling locomotion on a frictional surface or granular medium [18–20] (figure 1). In such a dissipation-dominated environment, drag is well described by the resistive force theory (RFT), which only considers local tangential and perpendicular drag coefficients. Here, we consider the resistive forces based on a viscous response of a Newtonian medium, as opposed to models based on Coulomb friction [23], such that the local force depends linearly on the velocity of the body section. This approach is known to be widely applicable across all scales in highly dissipative systems [19,20,24–27], with great accuracy for small deformations, that may decrease when non-local effects play an important role.

In the highly dissipative and inertialess regime, kinematic reversibility of the system necessitates non-reciprocal deformation of a self-propelled object to achieve net locomotion, which is well known as the scallop theorem [28,29] in low-Reynolds-number hydrodynamics. For instance, a complete in-phase synchronization of motor activation of ipsilateral muscles leads to a reciprocal deformation; hence, the animal should break the reciprocity of the internal states. In contrast, some fish can still swim in this case, a gait known as oscillatory swimming at large Reynolds numbers. Consequently, non-reciprocity in dissipative media imposesspecific constraints on motor activity.

A strategy to overcome the reciprocity constraint would consist of coupling CPG dynamics with the fluid response to body motion through sensory feedback to produce elementary motor patterns for propulsion. Such a scenario could be exploited by microswimmers such as nematodes like *Caenorhabditis elegans*, although experimental evidence remains partial. Recent studies suggest the existence of a CPG-like network in *C. elegans*, reporting multiple sections of rhythmic cycles [30], and a relaxation-oscillator model supports these experimental findings [31].

The rhythmic neural activity of *C. elegans* has been used to develop integrated neuromechanical models for its undulatory locomotion [32,33]. More recently, based on RFT, fluid–structure interactions have been incorporated into models that contain both muscle activation and neural coupling with proprioceptive feedback. These models have successfully reproduced gait transitions of *C. elegans* between swimming and crawling and waveform modulation for swimming in media with different viscosities [34,35]. Johnson *et al*. [35] further theoretically derived a coarse-grained model from their integrated model by phase-oscillator reduction and found that the reduced model captures well the gait adaptation of swimming in *C. elegans*, motivating a simplified and minimal mathematical model for animal gait adaptation in dissipative media.

Hence, in this study, we develop a simple and widely applicable algorithm for neuromechanical adaption to different environments in a dissipation-dominated regime, by using local exteroceptive mechanical feedback from the environment. To do so, we extend the coupled-oscillator model with fluid–structure interactions used in [9], which reported robust self-organized locomotion through local hydrodynamic sensing in the inertial regime.

Our primary aim is to understand the mechanisms of neuromechanical adjustment and the complexity of animal behaviour in a dissipation-dominated regime through a simple and minimal mathematical model. Therefore, we introduce a high-level model of motor activation taking the form of CPGs that produce reciprocal motion in the absence of external stimuli. The underlying motivation is to introduce a fundamental motor control that does not favour any particular propulsive gait, due to motion reciprocity. By introducing sensory feedback in this specific CPG, we show that locomotion can be an emerging property of couplings between the body dynamics and the neuromotor system in dissipative media. Indeed, we report various motions, such as crawling and swimming at low and high viscosity, resulting from the adaptation to mechanical properties of the swimmer or drag of the dissipative medium.

To describe the diversity of the observed motions, we take inspiration from the behavioural-state space representation of *C. elegans*, recently introduced in an experimental study [36]. In this framework, its behaviour can be fully characterized by the state of its mechanical system and body shape. Our second aim is to analyse and design complex behaviours in terms of smooth trajectories produced by our neuromechanical model.

To reproduce behavioural variability, we hypothesize that this smooth trajectory is ruled by a non-autonomous dynamical system displaying different attractors. Here, the gait is represented by a periodic orbit or limit cycle. We show how couplings between body-environment dynamics and CPGs can generate transient dynamics between stable and unstable periodic orbits, which can reproduce the reversal of the motion direction. Motivated by the turning motion of *C. elegans*, we also introduce a phase-locking mechanism in the CPG to create a local fixed point in the state space corresponding to a posture producing the omega-turn manoeuvre [37]. Beyond the case of locomotion in *C. elegans*, our aim is to present a general theoretical framework to describe the emergence of complex motor patterns.

The content of the article is as follows: §2 introduces our mathematical model of a slender elastic object in a dissipative medium. The inner muscle activity is driven by the rhythmic pattern generated by CPGs, and the CPG phase is described by a coupled oscillator, which is locally modulated through mechanosensory feedback. We present typical emergent behaviours in §3 and further analyse the dynamical system with a focus on the trajectories in a Poincaré section in §4. In §5b, by controlling the phase dynamics, we evaluate a manoeuvring algorithm that reproduces omega turns of *C. elegans*. Concluding remarks are made in §6.

The Matlab code used for all numerical simulations conducted in this article is freely available [38].

## 2. Model

### 2.1. Body–environment coupling

We model the animal's body as an inextensible elastic slender rod of length L. The centreline is denoted by its arclength s∈[0,L], and its position by 𝒙(s,t). To calculate the force and torque balance for the local rod segment, contributions from elasticity, drag from the environment and inner activation are incorporated.

**Figure 2.**
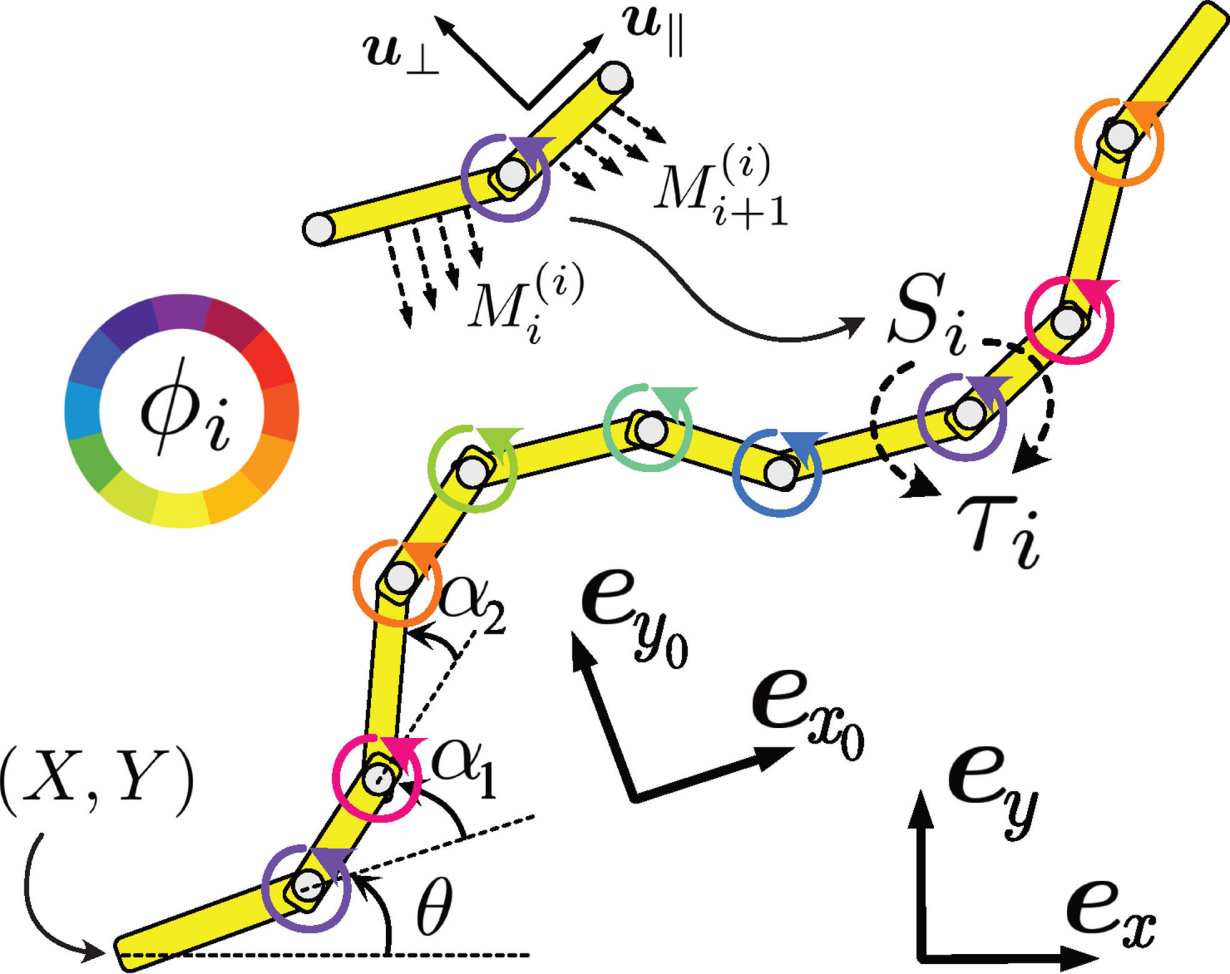
Schematic of the model and the two frames of reference. A slender elastic rod is represented by N links connected by elastic hinges. Each hinge generates internal torque $\tau_{i}$(i=1,2,…,N−1), as a function of the phase $\phi_{i}(t)$, which models the local CPGs. The phase dynamics are described by a coupled-oscillator equation with modulation by local signal $S_{i}(t)=M_{i}^{\left(i\right)}+M_{i+1}^{\left(i\right)}$, with $M_{i}^{\left(j\right)}$ being the drag on the ith link around the jth hinge (inset). The laboratory-fixed frame $\left\{{𝒆}_{x},{𝒆}_{y},{𝒆}_{z}\right\}$ and body-fixed frame $\left\{{𝒆}_{x_{0}},{𝒆}_{y_{0}},{𝒆}_{z_{0}}\right\}$ are introduced, and the motions are assumed to be restricted in the *x*–*y* plane. The motion of the object is represented by the end of the first link (X,Y) and its angle to the laboratory frame θ. The shape of the body is described by N−1 relative angles $\alpha_{i}$.

We assume planar locomotion (we set this as our xy plane) and no twisting motion. In biological locomotors, muscular activation usually occurs through the actuation of several elements along the body. For instance, *C. elegans* has approximately six muscular modules on its body [35]. This discrete activation structure within a continuous elastic rod is conveniently described by the coarse-grained representation of the elastic slender rod, known as the N-link model, where the object is represented by N equal-length links connected at N−1 hinges [39,40] (figure 2). Here, we take N=10, following a previous study [9]. We write the position of the end of the first link as (X,Y) and the angle from the x-axis as θ. As shown in figure 2, these represent the position and orientation of the body and define the body-fixed frame $\left\{{𝒆}_{x_{0}},{𝒆}_{y_{0}},{𝒆}_{z_{0}}\right\}$, which is distinct from the laboratory frame $\left\{{𝒆}_{x},{𝒆}_{y},{𝒆}_{z}\right\}$. The object shape is described by N−1 relative angles between the neighbouring links and denoted by $\alpha_{i}$ (i=1,2,…,N−1).

For the body-environment coupling, we use the RFT, in which the local force per unit length at 𝒙 is assumed to be proportional to the local tangent and normal velocities, ${𝒖}_{∥}$ and ${𝒖}_{⟂}$, as ${𝒇}^{\text{env}}=-c_{∥}{𝒖}_{∥}-c_{⟂}{𝒖}_{⟂}$, where the coefficients, $c_{∥}$ and $c_{⟂}$, are the drag coefficient in each direction (figure 2 inset). Since the drag force by the environment is linearly dependent on the local velocity, to represent the local velocity, we need linear and angular velocities as well as deformation velocity, for which we introduce a generalized velocity of the moving and deforming body as $\dot{z}=({\dot{X}}_{0},{\dot{Y}}_{0},\dot{\theta },{\dot{\alpha }}_{1},\ldots ,{\dot{\alpha }}_{N-1}{)}^{\text{T}}$, where the dot symbol indicates the time derivative and $\left({\dot{X}}_{0},{\dot{Y}}_{0}\right)$ is the velocity vector in the body-fixed frame.

For inertialess motion, the dynamical system takes the form of a linear system between the generalized velocities and the internal torques, thanks to the generalized resistance matrix 𝐀(𝜶) [41]. This linear relationship is based on the proportionality between drag and generalized velocity, with coefficients depending on the instantaneous shape 𝜶. The matrix 𝐀(𝜶) is obtained by imposing balance of forces and torques, which includes drag and internal torques, on the whole body as well as on each junction, thus, giving N+2 linear equations coupling the generalized velocities to the internal torques. Detailed expressions are provided in [39]. We then arrive at a matrix form of the body–environment coupling as [42,43]:


(2.1) $$\begin{array}{ccc} & 𝐀\dot{𝒛}=𝒕 & \end{array}$$

The vector 𝒕 on the right-hand side is the generalized force and torque vector, and for free locomotion only the bottom N−1 rows may have non-zero values as $𝒕=(0,0,0,\tau_{1},\ldots ,\tau_{N-1}{)}^{\text{T}}$. Here, the torque at the ith hinge contains elastic bending and internal actuation, which we denote $\tau_{i}=\tau_{i}^{\text{ela}}+\tau_{i}^{\text{int}}$.

### 2.2. Coupled oscillators and mechanosensation

We assume that the inner activation is controlled by a network of interconnected neural oscillators forming the CPGs, which provide rhythmic motions to the junctions. To model the cyclic activation of the junctions, we assume that the ith oscillator is associated with the corresponding hinge, and generates a periodic activation signal given by a phase denoted as $\phi_{i}(t)$(i=1,2,…,N−1) (figure 2).

To model the elasticity of the body, we use the Euler–Bernoulli constitutive relation, which assumes a linear relation between the bending moment and local curvature. With a bending modulus κ for the discretized rod, we then have $\tau_{i}^{\text{ela}}=-\kappa \alpha_{i}$. For torque actuation, we simply assume a periodic sinusoidal mode [9], given by $\tau_{i}^{\text{int}}=\tau \;\cos\;\phi_{i}(t)$, where τ is the torque actuation strength at each hinge. The torque function is then summarized as $\tau_{i}=-\kappa \alpha_{i}+\tau \;\cos\;\phi_{i}(t)$.

Following previous studies of CPGs for undulatory locomotion [44,45] to describe this system, we describe the oscillator dynamics by a limit cycle close to a Hopf bifurcation. In this case, phase reduction of the dynamics leads to [46]


(2.2) $$\begin{array}{ccc} & \dot{\phi_{i}}=\omega_{0}+\sum_{j}C_{ij}\sin(\phi_{j}-\phi_{i})+Z(\phi_{i})S_{i}(t), & \end{array}$$

where we consider a symmetric, nearest-neighbour phase coupling with $C_{i,i\pm 1}=C$ and zero otherwise. The phase oscillators are assumed to have a common intrinsic frequency $\omega_{0}$ and coupling with their neighbours with a common strength that is modulated by sensory feedback from the surrounding mechanical environment. Close to a Hopf bifurcation, the phase sensitivity function takes the form of $Z(\phi_{i})=\sigma \;\cos\;\phi_{i}(t)$ with a sensitivity strength σ [9].

The phase sensitivity function $Z(\phi_{i})$ characterizes the response of a limit cycle weakly perturbed by the sensory feedback $S_{i}(t)$. The function $Z(\phi_{i})$ modulates the input signal $S_{i}(t)$ such that their correlation modifies the phase dynamics, resulting in-phase acceleration or deceleration. Synchronization and phase locking between these two signals is an emergent phenomenon that depends on both the choice of the sensitivity function and the nature of the sensory feedback.

As for the sensory feedback, we follow previous literature on biological exteroception [9,47,48]. In their model for rhythmic contraction of true slime mould, Kobayashi *et al*. [47,48] considered local peristaltic pumping generated by phase oscillators and used the local hydrodynamic pressure as exteroceptive feedback. In the study of Tandiackal *et al*. [9], in which exteroceptive feedback in fish-like robot is studied, the authors consider local hydrodynamic forces at the actuation point on both sides of the elongated body and use the difference between the forces on each side as the exteroceptive signal. In our model, taking the slender limit of this difference, we employ the local torque load at each hinge as the exteroceptive feedback. Let $M_{i}^{\left(j\right)}$ be torque (per unit length) from the environmental drag on the ith link around jth hinge, or equivalently,


(2.3) $$\begin{array}{ccc} & M_{i}^{\left(j\right)}={𝒆}_{z}\cdot \int_{s_{i-1}}^{s_{i}}\left(𝒙(s)-𝒙(s_{j})\right)\times {𝒇}^{\mathrm{env}}(s)\;\frac{ds}{\Delta s}, & \end{array}$$

where Δs=L/N and $s_{i}=i\Delta s$ corresponds to the position of the ith hinge (inset of figure 2). We then set a fore–aft symmetric feedback $S_{i}(t)=M_{i}^{\left(i\right)}+M_{i+1}^{\left(i\right)}$, which is the local torque load on the ith hinge.

Other choices to model proprioceptive sensory feedback include using the local curvature as signal input, which, however, must be asymmetric to break the fore–aft symmetry and generate undulatory locomotion. Some mathematical models consider asymmetric and non-local signal inputs with weight on the posterior neighbour (e.g. [35]), enforcing a directed motion. In contrast, our exteroceptive feedback is a local and symmetric function, which fits our purpose to simplify the mechanism for adaptive locomotion.

### 2.3. Non-dimensionalization and model parameters

The mechanical torque on the body is calculated through the RFT, which is applied for various undulatory motions in dissipation-dominated environments, such as low-Reynolds-number flows, gel-like structures and mud and sand. We then introduce the anisotropic drag ratio as $\gamma =c_{∥}/c_{⟂}$, which is known to affect locomotion. Following the data obtained for *C. elegans*, we use γ=1/2 for *swimming* and γ=1/70 for *crawling* [25,26]. When the model swims in a low-Reynolds-number Newtonian fluid, the coefficients are theoretically estimated [49] as $c_{⟂}=4\pi \mu /[\log(2L/d)]$ in a slender asymptote, where μ is the fluid viscosity and d is the cross-sectional radius of a slender body.

For our numerical and theoretical analyses, we non-dimensionalize the above system by setting the scales L=1 for length, $T=2\pi /\omega_{0}=1$ for time, and κ=1 for force. An important non-dimensional number of the system is the so-called ‘sperm number’, which represents the effective flexibility of an oscillatory elastic rod in a highly damped system, defined as $\text{Sp}=L(\omega_{0}c_{⟂}/\kappa_{b}{)}^{1/4}$ [39], where $\kappa_{b}=\kappa /(L/N)$ is the bending modulus. For flagellated microswimmer, a typical value of Sp for human sperm flagella in water is Sp≈4 [50]. The estimation of Sp for *C. elegans* locomotion; however, is limited due to estimated values of the bending modulus, which could vary by up to two orders of magnitude depending on experiments [35].

Sznitzman *et al*. [51] estimated the bending modulus as $\kappa_{b}\approx 4.2\times 10^{-16}\text{N}\cdot {\text{m}}^{2}$ and observed the beating frequency 1/T≈2.4 Hz in watery medium ($\mu \approx 10^{-3}\text{Pa}\cdot \text{s}$). With these parameters and $L=10^{-3}\text{m}$ for the body length, we may estimate the value of sperm number as Sp≈2.4. Fang-Yen *et al*. [52], however, estimated the bending modulus as $\kappa_{b}\approx 9.5\times 10^{-14}\text{N}\cdot {\text{m}}^{2}$ observe the beating frequency 1/T≈1.8Hz. With these parameters, the sperm number is estimated as Sp≈0.7. Fang-Yen *et al*. [52] also examined *C. elegans* swimming in viscous medium. From their observations, one may estimate the value of sperm numbers as Sp≈5.2 for the medium $10^{3}$-fold more viscous than water with 1/T=0.5Hz, and as Sp≈12.5 for the $10^{4}$-fold viscous medium with 1/T=0.15Hz. Shen *et al*. [53] evaluated the drag coefficient of *C. elegans* crawling on agar gels, suggesting an increase of drag coefficient by circa 400 times than that of swimming in water. We may then estimate the sperm numbers for crawling ranging Sp=4.1−16.0 by considering the decrease of the beat frequency in crawling (1/T≈0.5Hz). In this article, to cover a biologically relevant range, we consider a range of Sp=1−12. Other parameters are drag coefficient ratio γ, non-dimensionalized actuation strength $\hat{\tau }$, coupling strength $\hat{C}$ and sensitivity strength $\hat{\sigma }$ using the same physical units of L, T and κ. Hereafter, we omit the hat symbol for the non-dimensionalized strengths.

## 3. Robust undulatory locomotion

### 3.1. Breakdown of motion reciprocity

**Figure 3.**
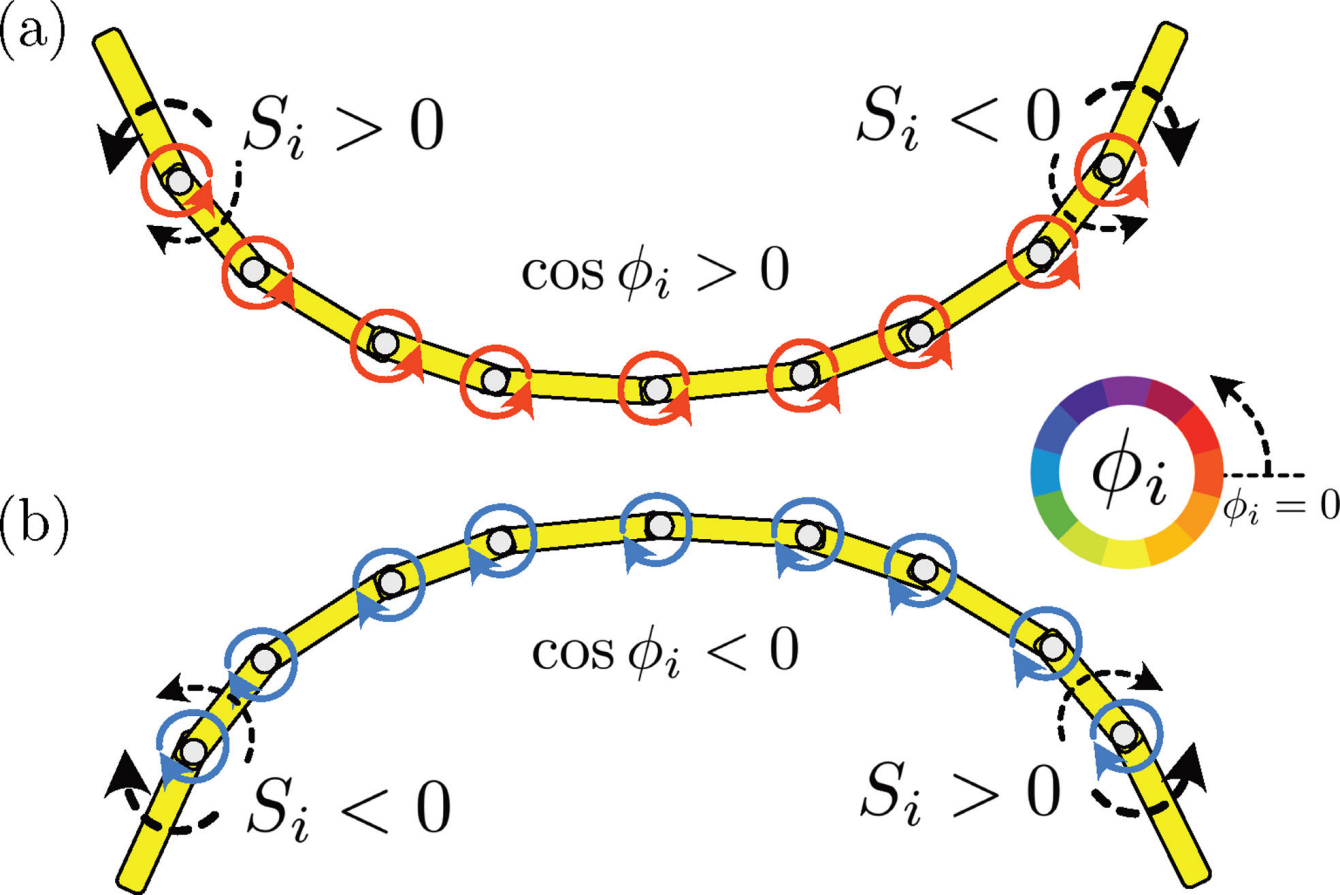
Schematic mechanism of symmetry breaking through mechanosensory feedback. Fully synchronized situations are illustrated with (*a*) $\cos\;\phi_{i}>0$ and (*b*) $\cos\;\phi_{i}<0$. The direction of the local torque actuation is opposite, and the shape becomes concave in (*a*) and convex in (*b*). Despite the uniform distribution of the torque, the local torque load is no more uniform along the object, yielding fore–aft asymmetric phase modulation due to mechanoreception. In both phases, the product of $S_{i}$ and $\cos\;\phi_{i}$ is always positive at the left end and negative at the right end. Thus, when the sensitivity strength, σ, is positive, the phases are accelerated at the left and decelerated at the right, leading to a travelling undulatory motion.

Before proceeding to the simulation results, we first consider how motion non-reciprocity can be produced by breaking the symmetry with respect to the locomotor’s transverse plane in the oscillator chain from the fore–aft symmetric sensory feedback function. The schematic in figure 3 explains the symmetry-breaking mechanism. In figure 3*a*, the oscillators are fully synchronized with $\cos\;\phi_{i}>0$ such that the anticlockwise torque generates a concave shape, where the end links rotate more quickly. The local torque load is therefore anticlockwise at the left end $\left(S_{i}>0\right)$, while the torque load is clockwise at the right end $\left(S_{i}<0\right)$, yielding a negative gradient of resistive torque along the rod. This gradient is then projected along the oscillator through the correlation $\cos(\phi )S_{i}$ in the phase equation (2.2).

When the synchronized phases are satisfied $\cos\;\phi_{i}<0$ (figure 3*b*), the direction of the local torque actuation reverses and the shape becomes convex. As in figure 3*b*, the local torque load has the opposite sign in the left and right ends but with the opposite sign of $S_{i}$ for the case of figure 3*a*, that is, a positive gradient of $S_{i}$. Nonetheless, the modulation to the phase dynamics, a product of $S_{i}$ and $\cos\;\phi_{i}$, remains positive at the left end and negative at the right end in both phases. Thus, when the sensitivity strength, σ, is positive (negative), the phases are accelerated on the left (right) side of the object, and decelerated on the right (left) side of the object, leading to symmetry breaking at the origin of the travelling wave of motor activation.

### 3.2. Robust undulatory locomotion

We now present simulation results of our model, equations (2.1) and (2.2). We have numerically calculated by an explicit time integration with the variable order method, implemented by a MATLAB built-in solver (ode15s).

We evaluated the simulation results in a different medium by changing the sperm number Sp and torque actuation strength τ for swimming dynamics with γ=1/2. In a wide parameter regime, we found that the object eventually exhibits stable undulatory locomotion by breaking the fore–aft symmetry (figure 3). The direction of motion is governed by the sensitivity strength sign, and we found forward motion (moving towards the first link) when σ>0 and backward motion (towards the last link) when σ<0.

**Figure 4.**
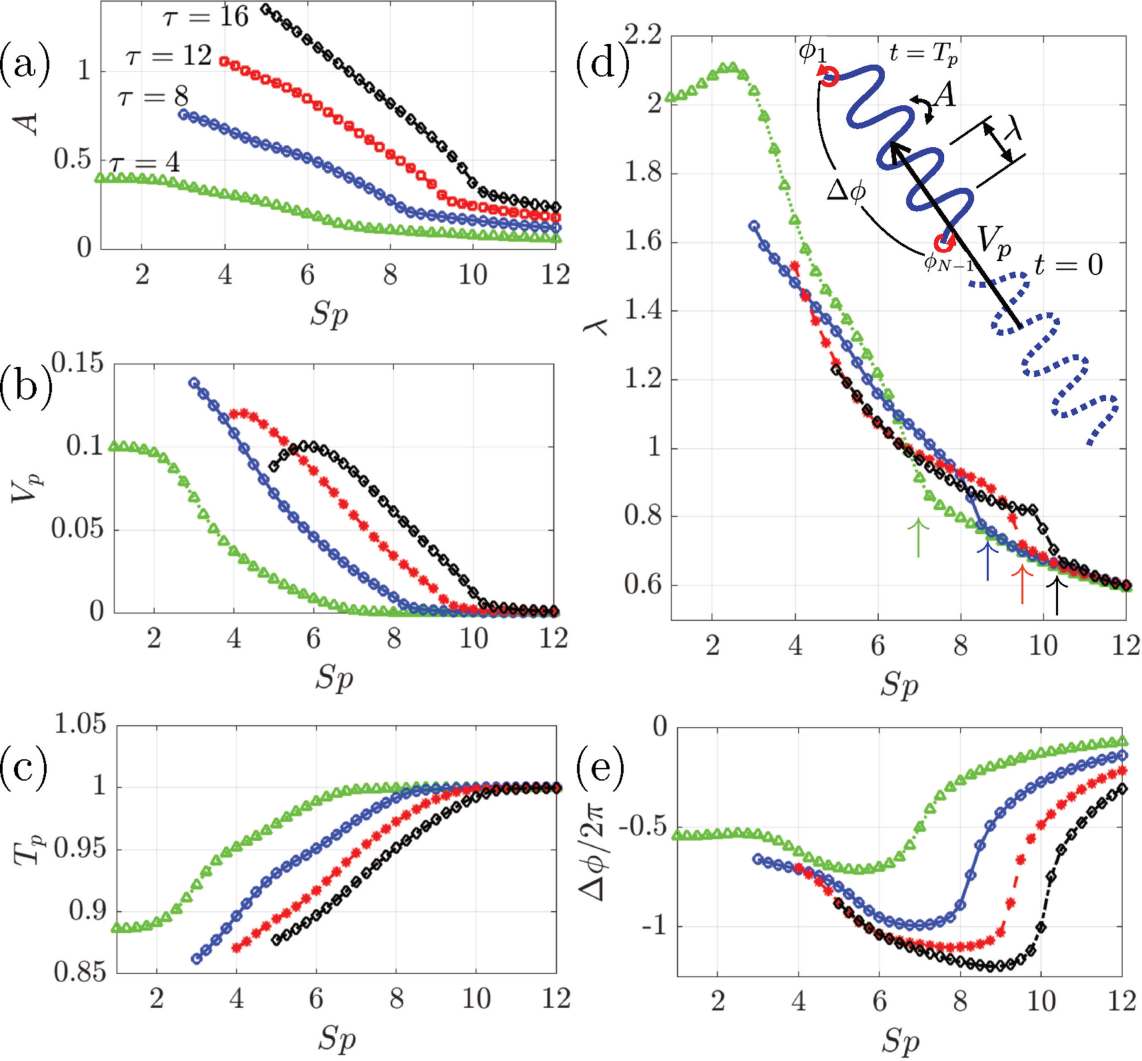
Shape morphology and motion parameters of emergent undulatory locomotion in different Sp with 1≤Sp≤12 and actuation strength τ∈{4,8,12,16}. (*a*) Mean maximum angle, A, (*b*) mean velocity, $V_{p}$, (*c*) motion frequency, $T_{p}$, (*d*) wavelength, λ and (*e*) head-to-tail phase difference Δϕ. Schematics of the parameters are shown in the inset of (*d*). With a small Sp and larger actuation, data are not shown due to non-physical large amplitude with body’s self-intersection. The arrows in (*d*) show critical values of Sp that exhibit sudden drops of λ.

Obtained shape morphology and motion parameters of emergent undulatory locomotion are summarized in figure 4. Other model parameters are fixed as C=1, σ=8.

We present mean maximum angle between links, A, mean velocity, $V_{p}$, motion frequency, $T_{p}$, wavelength, λ and head-to-tail phase difference Δϕ. Each parameter is schematically shown in the inset of figure 4*d*.

As shown in figure 4*a*, the values of A, which measures the maximum curvature and amplitude of the undulation, increases as τ increases, while these values decrease at a higher Sp because of a larger viscous drag force. Some data at a small Sp are not obtained due to non-physical large amplitude with collision between links.

The mean velocity decreases at a higher Sp and drops to almost zero at a value of Sp ranging between 6 and 10 depending on τ (figure 4*b*). These critical values of Sp are clearly shown by a sudden drop of wavelength shown by arrows in figure 4*c*. Above these critical values of Sp, the coherent waves cease and locomotive behaviours are no longer observed. These changes of regime are reflected in saturation of $T_{p}$ around $T_{p}\approx 1$ in figure 4*c* and sudden change of the phase difference, |Δϕ|, in figure 4*e*.

For a fixed internal actuation τ, we have found there is a wide range of Sp with stable undulatory swimming but with a maximum Sp. To swim in a high Sp region, one needs a larger actuation. Interestingly, this tendency of larger actuation in higher viscosity is compatible with *C. elegans* swimming [52] and human sperm swimming [54,55]. Moreover, the wavelength of undulatory swimming at a higher viscosity is known to decrease in these species, which also qualitatively agrees with the current results.

For constant wavelength, we should observe |Δϕ|=2π/λ, which is almost true for small Sp with coherent undulatory waves. However, this does not hold when the coherency disappears for larger Sp, since the net phase lag no longer reflects the wavelength of body deformation.

The shape morphology and motion parameters for crawling motions with γ=1/70 are qualitatively the same as the swimming motion of figure 4 with a motion decoherency at a large Sp accompanied by decrease of wavelength (see electronic supplementary material, figure S1). The dependence of other parameters such as C and σ, and γ are also shown in electronic supplementary material, figures S2–S4, showing robust emergence of undulatory motion in a wide parameter regime. In particular, decreasing γ in general leads to a larger undulatory motion wavelength in crawling (electronic supplementary material, figure S4).

**Figure 5.**
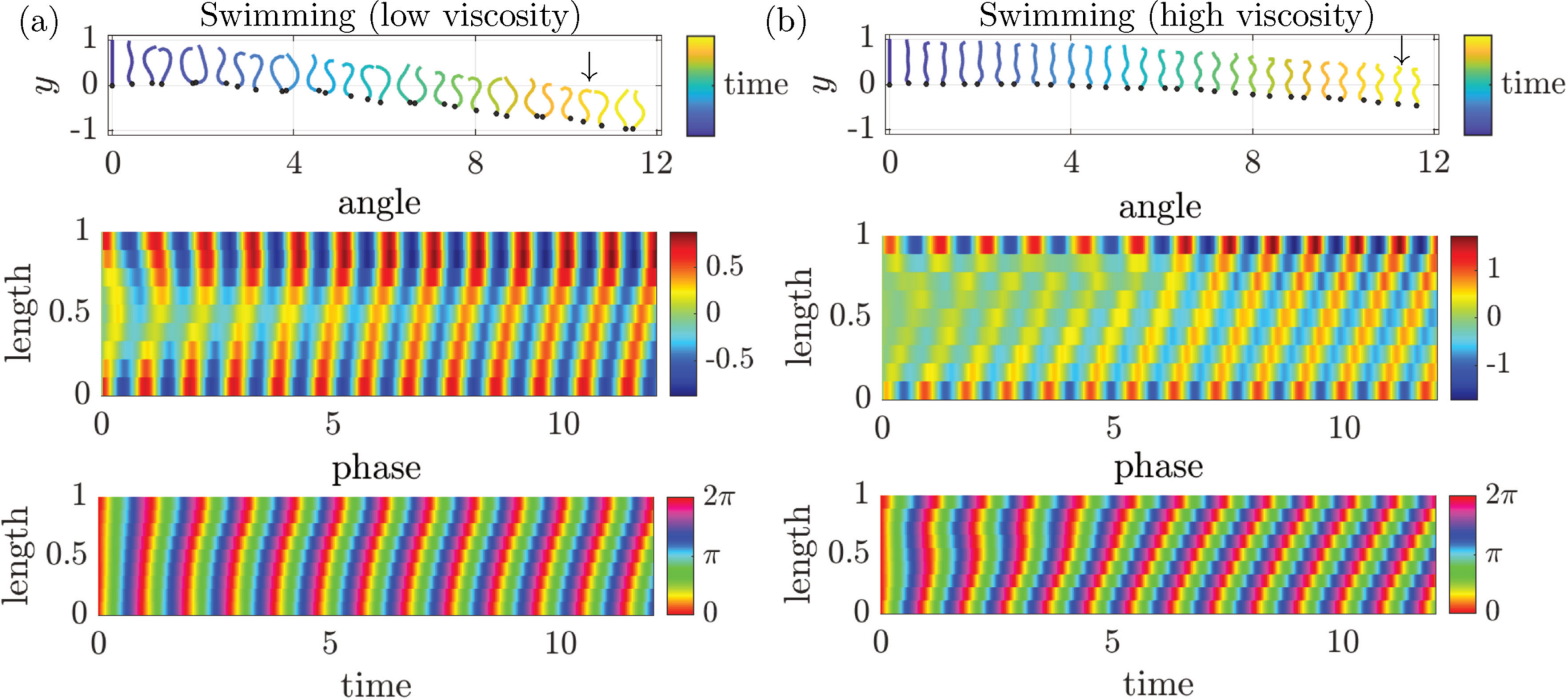
Typical emergent motions of (*a*) swimming in a lower viscosity medium (Sp=4) and (*b*) swimming in a higher viscosity medium (Sp=8). The model animal was initially aligned in a straight configuration with the same phase. The top panels show the time series of shape gaits. The shape of the locomotor is plotted in different colours to present the time evolution and the black dot denotes the end of the first link in each snapshot. The time series of the angle $\alpha_{i}$ and the phase $\phi_{i}$ are shown as kymographs in the middle and bottom panels. The model parameters are (*a*) Sp=4, τ=8, σ=4, C=1 and (*b*) Sp=8, τ=16, σ=12, C=1. The resulting motion parameters of undulatory locomotion are (*a*) $A=0.64,\lambda =1.67,V_{p}=0.109,T_{p}=0.952,\Delta \phi /(2\pi )=-0.63$, (*b*) $A=0.95,\lambda =0.82,V_{p}=0.065,T_{p}=0.907,\Delta \phi /(2\pi )=-1.31$. See also electronic supplementary material, Videos S1-S2.

In figure 5*a*, we show typical swimming dynamics (γ=1/2) in a fluid with a lower (Sp=4) and higher (Sp=8) sperm numbers (see also electronic supplementary material, Videos S1-S2). The top panel shows snapshots of the shape of the swimmer in different colours. The end of the first link in each snapshot is denoted by a black dot for illustrative purposes. The model animal, initially aligned in a straight configuration, starts to create a travelling wave and eventually exhibits stable periodic swimming. The middle and bottom panels present the kymographs of the angle $\alpha_{i}(t)$ and the phase $\phi_{i}(t)$. The other parameters were set as (a) τ=8, σ=4 and C=1 and (b) τ=16, σ=12.

**Figure 6.**
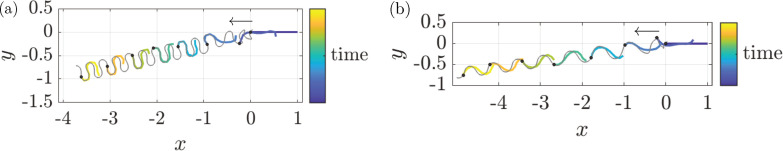
Typical emergent motions of crawling on an agar gel. The model animal was initially aligned in a straight configuration with the same phase. Snapshots of the shape in the xy plane are superimposed on the trajectory of the end of the first link. The shape of the locomotor is plotted in different colours to present the time evolution and the black dot denotes the end of the first link in each snapshot. The model parameters are (*a*) Sp=8, τ=16, σ=12, C=1 and (*b*) Sp=6, τ=8, σ=12, C=0.1. The resulting motion parameters of undulatory locomotion are (*a*) $A=1.16,\lambda =1.25,V_{p}=0.288,T_{p}=0.892,\Delta \phi /(2\pi )=-0.78$, (*b*) $A=0.76,\lambda =0.97,V_{p}=0.501,T_{p}=0.915,\Delta \phi /(2\pi )=-1.08$. See also electronic supplementary material, Videos S3-S4.

We then focus on crawling behaviour by setting the drag anisotropy ratio to γ=1/70 [26]. Example crawling motions are shown in figure 6 and electronic supplementary material, videos S3–S4. Of particular note, the locomotion trajectory begins to follow the body shape, which is seen in *C. elegans* [56]. In figure 6, we plot superimposed snapshots in the physical x−y space to show the shape and the trajectory of the end of the first link, which is again denoted by a black dot in each snapshot. The parameter set used in the figure was Sp=8, τ=16, σ=12, C=1 in (a) and Sp=6, τ=8, σ=12, C=0.1 in (b).

## 4. Stability of periodic orbits

In the previous section, stable, robust periodic locomotion was observed in a wide range of parameters. We now seek mathematical characterization of the existence and stability of these emerging periodic orbits, by adopting a dynamical system viewpoint.

**Figure 7.**
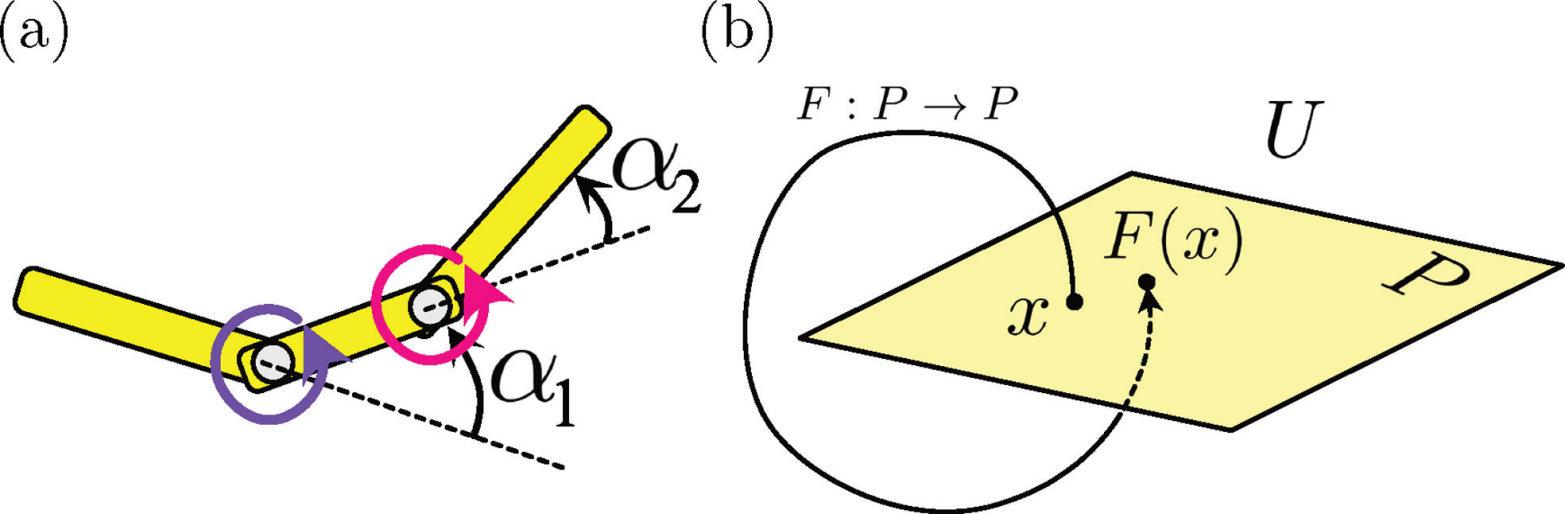
(*a*) Schematic of Purcell’s three-link model. (*b*) Schematic of Poincaré section P in the state space U and the associated Poincare map F:P→P.

We introduce the dynamics state for a generic N-link model as


(4.1) $$\begin{array}{ccc} & 𝒙=(\alpha_{1},\alpha_{2},\ldots ,\alpha_{N-1},\phi_{1},\phi_{2},\ldots \phi_{N-1}{)}^{\text{T}}\in U, & \end{array}$$

where $U={\mathbb{R}}^{N-1}\times {𝕋}^{N-1}$ is the state space. We choose our Poincaré section at $\phi_{1}=0$ and denote the section as $P={\mathbb{R}}^{N-1}\times {𝕋}^{N-2}$, which leads to the associated Poincaré map F:P→P (figure 7*b*). Here, we consider a periodic orbit in the state space including its configuration and CPG phases, while the CPG phase is a coarse-grained representation of the oscillatory neural dynamics at each junction.

A fixed point ${𝒙}^{*}$ on the Poincaré section corresponds to a periodic orbit in the state space, or in other words, the periodic animal morphology, including undulatory locomotion. To detect periodic orbits in U, which is regarded as a fixed point in P such that $F({𝒙}^{*})={𝒙}^{*}$, we use the standard Newton–Raphson method through ${𝒙}_{m+1}={𝒙}_{m}-[𝐉({𝒙}_{m}){]}^{-1}[F({𝒙}_{m})-{𝒙}_{m}]$ for an integer m, where $𝐉({𝒙}_{m})$ represents the Jacobian matrix. The eigenvalues of the Jacobian matrix approximate the Floquet exponents when the algorithm converges by iteration. For methodological details, one may refer to a review paper [57] and applications in sperm swimming dynamics [58].

### 4.1. Purcell’s three-link model

We first consider the case of N=3, where the three links are connected by two hinges (figure 7*a*). This is Purcell’s three-link model [28], which has been widely used as a simple and canonical mathematical model of microscale swimming [42,59]. The state space U is then four-dimensional and the Poincaré section P is three-dimensional.

Before proceeding to the numerical results of the periodic orbits, let us first analyse the condition under which the periodic orbits exist. When N=3, the phase dynamics are written as


(4.2) $$\begin{array}{ccc} & \left\{ \begin{array}{cc} {\dot{\phi }}_{1} & =\omega_{0}+C\;\sin(\phi_{2}-\phi_{1})+\sigma \;\cos(\phi_{1})S_{1}(t)\\ {\dot{\phi }}_{2} & =\omega_{0}+C\;\sin(\phi_{1}-\phi_{2})+\sigma \;\cos(\phi_{2})S_{2}(t) \end{array}\right., & \end{array}$$

and by introducing the phase difference $\Delta \phi =\phi_{2}-\phi_{1}$, we obtain the phase equation:


(4.3) $$\begin{array}{ccc} & \Delta \dot{\phi }=-2C\;\sin(\Delta \phi )+\sigma \left[\;\cos(\phi_{2})S_{2}-\cos(\phi_{1})S_{1}\right]. & \end{array}$$

In the absence of mechanosensory feedback, namely, σ=0, a complete in-phase synchronization, Δϕ=2nπ (n=0,±1,±2,…), is a stable fixed point, while a complete anti-phase synchronization, Δϕ=(2n−1)π, is an unstable fixed point.

When the mechanosensory feedback is activated (σ≠0), two synchronized states can still be observed, which correspond to the in-phase and anti-phase synchronized states when σ=0, and they inherit their respective stability properties. In the small-amplitude case (τ≪1), we may analytically prove that these two synchronized states always exist, regardless of the values of Sp,γ,τ,σ and C. The detailed derivations are provided in the electronic supplementary materials.

**Figure 8.**
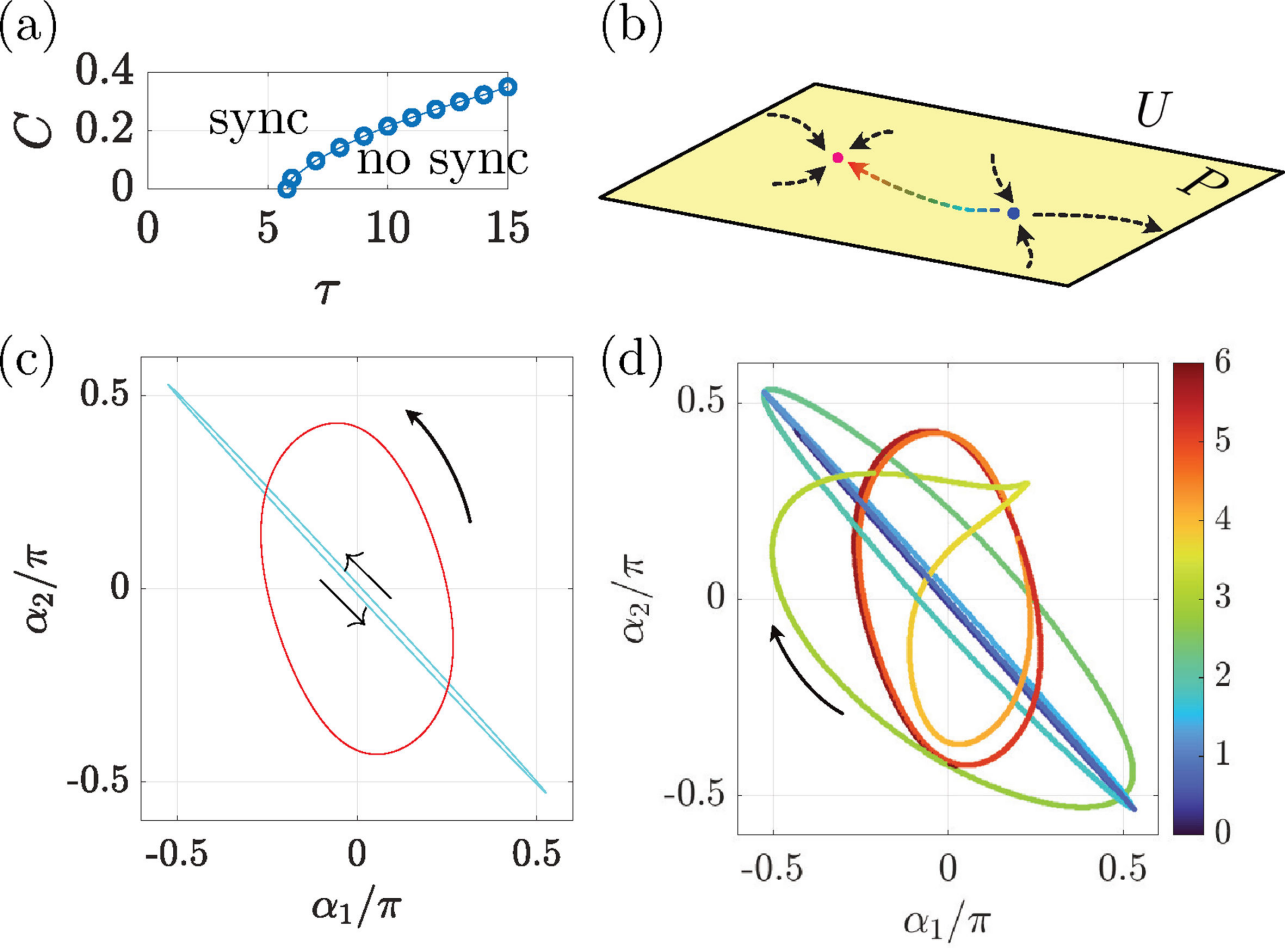
(*a*) Plots of critical value $C^{*}$ for different τ values for Purcell’s swimmer in a low-viscosity medium. (*b*) Schematic of the dynamical systems on Poincaré section P. There is one stable fixed point (red) and one saddle point (blue). (*c*) Periodic orbits of the three-link swimmers in the $\alpha_{1}-\alpha_{2}$ shape space. The large orbit (red) is stable, and the thinner orbit (blue) is unstable. Arrows indicate the direction of the trajectories. (*d*) Time evolution in the $\alpha_{1}-\alpha_{2}$ shape space, with the initial shape being set close to the unstable saddle. Different colours illustrate the time evolution. See also electronic supplementary material, video S5.

In the finite-amplitude case, however, the synchronized states can cease due to a saddle-node bifurcation. Indeed, there may be a critical coupling constant $C^{*}$, below which the synchronized states disappear. Figure 8*a* plots the values of the critical coupling constant for different sizes of τ with the other parameters being the same as in figure 5*a*. At a large amplitude with τ≳5.8, phase slip occurs in the parameter region without synchronized states.

Nonetheless, this weak coupling region is very limited. In a large parameter region, which specifically contains the typical parameters for biologically relevant motion, there is one stable periodic orbit (SPO) and one unstable periodic orbit (UPO). We established through numerical stability analysis that the UPO is a saddle point. Figure 8*b* shows a schematic of the structure of the dynamical systems in U and on P, where the SPO (the red point) and UPO (the blue point) are connected by heteroclinic orbits. By setting the parameter values as those of the low-viscosity swimmer in figure 5*a*, we numerically detect the SPO and UPO. Figure 8*c* plots them in the $\alpha_{1}-\alpha_{2}$ shape space, with red and blue orbits representing the SPO and UPO, respectively. When the initial shape is located near the UPO, the dynamics evolve along a heteroclinic orbit, as shown in figure 8*d*. As time progresses (from blue to red), the trajectory departs from the UPO and finally approaches the SPO with the cyclic direction changing from anticlockwise to clockwise, to anticlockwise in the $\alpha_{1}-\alpha_{2}$ shape space. Time evolution of shapes and trajectories in real space for figure 8*d* are found in electronic supplementary material, video S5.

### 4.2. Stable and unstable periodic orbits in N-link model: an example

We now proceed to the analysis of the generic N-link system with N>3. In this subsection, to illustrate the dynamical systems near a saddle-node bifurcation, we set up Sp=6 and τ=8 with a small C/σ value as C=0.1 and σ=12 with N=10. The emergent stable undulatory motions are shown in figure 6*b* and electronic supplementary material, video S4.

**Figure 9.**
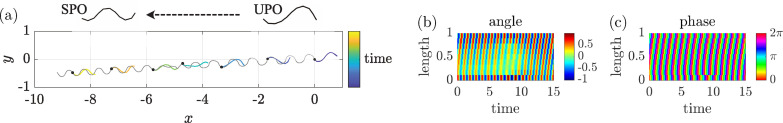
Transient crawling locomotion from an unstable saddle (UPO) to a stable limit cycle (SPO) with N=10. (*a*) Snapshots of the shape in the xy plane are superimposed on the trajectory of the end of the first link. The shape of the locomotor is plotted in different colours to present the time evolution and the black dot denotes the end of the first link in each snapshot. The object, initially located near the UPO (λ=1.27) in the state space, exhibits an eventual convergence to the SPO (λ=0.97) after transient behaviour. (*b,c*) Kymographs of the angle and phase dynamics, respectively. The model parameters are the same as in figure 6. See also electronic supplementary material, Video S6.

With the same numerical analysis of the Poincaré section, we detected a global SPO, by confirming that all the Floquet exponents have a negative real part. A snapshot of the shape gait is shown in the top left of figure 9*a*. In the same parameter regions, we also found a UPO. As is shown in the top right of figure 9*a*, the snapshot for this UPO shows a longer wavelength (λ=1.27) than that for the SPO waveform (λ=0.97). Other motion parameters are obtained as $A=0.61,V_{p}=0.667,T_{p}=0.938,\Delta \phi /(2\pi )=-0.72$. We confirm that one of the Floquet exponents is a positive real number, indicating that the UPO is a saddle, like in the Purcell three-link model.

Hence, if the model configuration is initially close to the UPO, the shape and phase dynamics evolve along the heteroclinic orbits that connect the SPO and UPO, as shown in figure 9. The waveform shifts from the unstable longer wave to the stable shorter wave. See also electronic supplementary material, video S6.

In the parameter set of figure 9, the critical coupling constant, below which synchronization ceases, is observed to be very small, at $C^{*}/\sigma \approx 0.01$. Furthermore, we found that large-scale stable locomotion emerges even for a larger N (≈100), suggesting that only one SPO exists even for a larger N.

In this section, we have seen a global attractor in the behavioural-state space, together with transient behaviour. In the next section, we will use this structural property to manipulate the locomotor dynamics.

## 5. Manoeuvring by modulating sensitivity

In the previous sections, we showed how our CPG-based model can achieve a stable locomotion pattern, with the mechanical feedback term playing a key role in the emergence of travelling waves. The strength of this term is measured by the sensitivity strength σ, which was observed to influence the gait aspect as well as the direction of motion depending on its sign. Hence, time variations of the sensitivity function are likely to allow changes in locomotion, such as a direction reversal or turn.

The existence of interneurons controlling forward and backward locomotion in *C. elegans* has been experimentally well-established [60]. This command neurons are numerically studied and demonstrated to switch between forward and backward motions [61]. The turning motion of *C. elegans* is also well-known to be driven by interneural commands [62], although the detailed neuromechanical mechanisms are still under investigation.

In this section, we explore the behaviours obtained when following this idea and considering σ as a function of time instead of a fixed parameter, taking inspiration from this *C. elegans* behaviour. The dynamical system governing the animal’s motion now appears as a non-autonomous system, and its trajectory can be guided by tuning σ as a control function.

### 5.1. Reverse motion

**Figure 10.**
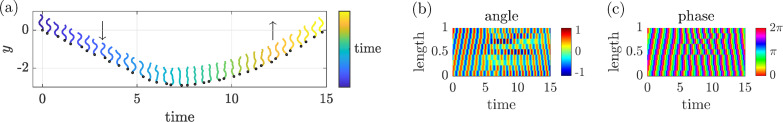
Transient crawling behaviour when the sign of signal strength σ switches. The SPO in figure 9 was used for the initial condition. We used the same parameter set except for σ, which was switched to −σ at time $t_{0}=0$. (*a*) Time series of the snapshots. The arrow indicates the movement direction, and the crawler moves in the −y direction until it turns to reverse to a backward motion in the +y direction. (*b,c*) Kymographs of the angle and phase dynamics, respectively. See also electronic supplementary material, video S7.

Sudden changes of the sensitivity value σ with time were reported in a neuromechanical model of *C. elegans* crawling motion as a plausible cause for motion reversal and turns [61].

From the point of view of the dynamical system, equation (2.2), a simple sign change of σ at some time $t_{0}$ modifies the dynamics landscape and in particular the position of the global SPO, which we know robustly emerges for both σ and −σ. Thus, forward–backward motion reversal naturally occurs upon this sensitivity change.

When the coupling strength C is sufficiently large, the locomotion immediately converges to the global SPO of the new dynamical system, and the crawler exhibits a quick reverse motion, which agrees well with *C. elegans* observations [37].

When the coupling weakens, the transient dynamics between the motion reversal exhibit more complicated behaviours, as shown in figure 10, where we present the transient behaviours of crawling locomotion with the same parameter set as in figure 9. We used the SPO of figure 9 as the initial configuration and changed the sign of σ as σ=12↦σ=−12 at time $t_{0}=0$. Snapshots of the shape gait are shown, with the horizontal axis indicating the time evolution. The arrow indicates the movement direction, and the crawler moves in the −y direction until it turns to a backward motion moving in the +y direction with a transient regime without phase synchrony (electronic supplementary material, video S7). As plotted in figure 10*b*,*c* the kymographs of the angle and phase dynamics also show complex transient dynamics until the angle reformulates the backward travelling wave.

### 5.2. Turning behaviour

So far, we have observed forward and backward motion, as well as the transition between both when the sensitivity strength σ is switched between a set value and its opposite. However, this abrupt switch can be modulated to allow manoeuvring and reaction to changes in the environment. This would particularly enable foraging strategies such as run-and-tumble or any kind of taxis.

To illustrate this, we take the omega-turn behaviour of *C. elegans* as an example and propose a way to reproduce it in our CPG model. The turning strategy of *C. elegans* on agar gels is called an ‘omega turn’ because it temporarily breaks the symmetry of its gait by curving towards one side in a shape that roughly mimics the Greek letter Ω [37,56].

To allow these occasional symmetry breaks within the CPG dynamics, we add a term in equation (2.2) governing the evolution of ϕ , which may reflect a neuronal command:


(5.1) $${\dot{\phi }}_{i}=\omega_{0}+\sum_{j}C\sin(\phi_{j}-\phi_{i})+\sigma_{1}(t)\cos(\phi_{i})S_{i}(t)+\sigma_{2}(t)K\cos(\phi_{i}-\psi_{i}),$$

where K and $\psi_{i}$ are set to induce the local angle $\alpha_{i}$ to curve into some angle $\alpha_{i}^{\Omega }$ and the amplitude of $\sigma_{2}$ measures this forcing strength. Details on how to design K and $\psi_{i}$ are provided in electronic supplementary material.

**Figure 11.**
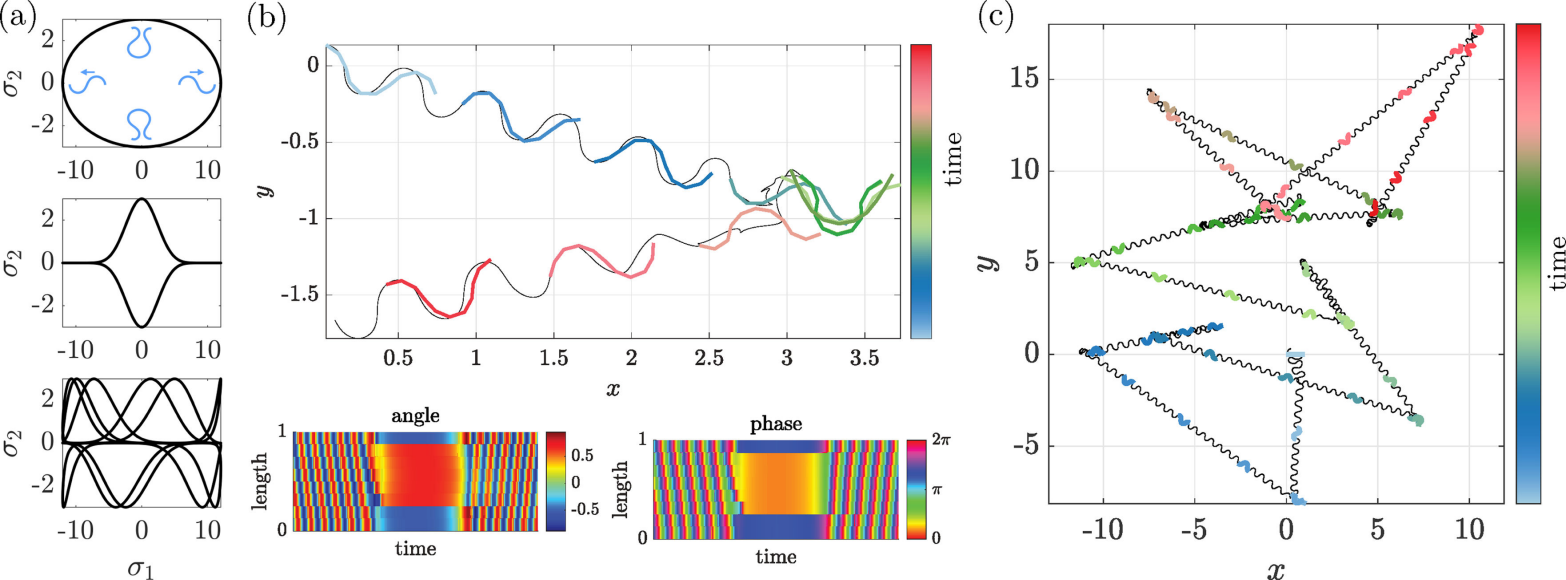
Simulated turning and foraging behaviour. (*a*) Representation of the control functions $\sigma_{1}(t),\sigma_{2}(t)$ in the $\left(\sigma_{1},\sigma_{2}\right)$ plane. The top panel summarizes the different states preferred by the model animal at each extremity of the activation pattern: (clockwise from the top) omega turn, forward motion, mirrored omega turn and backward motion. The middle and bottom panels, respectively, display the activation functions used to generate the trajectories of panels (*b*) and (*c*). (*b*) Detailed aspect of the omega-turn simulation, with associated angle and phase kymographs. (*c*) Long-time behaviour with decorrelated frequencies for $\sigma_{1}$ and $\sigma_{2}$, showing complex, irregular trajectories. See also electronic supplementary material, Videos S8-S9.

To clarify the role of the two activation functions in equation (5.1), the factor term of $\sigma_{1}$ sustains periodic locomotion as discussed previously, while the factor term in $\sigma_{2}$ drives each $\phi_{i}$ to converge to a constant value in a way that immobilizes the model animal in a given shape measured by target angles $\alpha_{i}^{\Omega }$. The relative amplitude of $\sigma_{1}$ and $\sigma_{2}$ determines which of these two behaviours dominate the instantaneous dynamics (electronic supplementary material, video S8).

With this set-up, when tuning $\sigma_{1}$ and $\sigma_{2}$ with respect to time, for example, following periodic functions, we may observe alternating forward or backward motion and also omega-turn-like behaviours effectively reorienting the animal’s direction of motion between forward and backward sections of the trajectory (top diagram of figure 11*a*), where we used the same parameter set of the crawling locomotion as in previous figures (electronic supplementary material, video S9).

A family of functions for $\sigma_{1}$ and $\sigma_{2}$ that may yield an interesting range of behaviours is given by


(5.2) $$\begin{array}{l} \left\{ \begin{array}{l} \sigma_{1}(t)=\sigma_{1}^{0}\cos^{2n_{1}+1}\left(\frac{t}{T_{1}}+\chi \right),\\ \sigma_{2}(t)=\sigma_{2}^{0}\sin^{2n_{2}+1}\left(\frac{t}{T_{2}}\right). \end{array}\right. \end{array}$$

The integers $n_{1}$ and $n_{2}$ should be relatively small to obtain the distributed activation, while large values simulate impulse-like behaviour. This latter option is suitable for the omega-turn activation. The corresponding activation function is plotted in the $\sigma_{1}-\sigma_{2}$ plane in the middle panel of figure 11*a*, and the resulting short-term trajectory is shown in figure 11*b*. The snapshots of the shape gait are illustrated in different colours from blue to green to red as time progresses. The model animal initially moves towards the right, but them briefly turns and then starts moving again towards the left.

This mechanism can be exploited to simulate the animal exploring its environment. Further variability may be obtained by desynchronizing the periodicity of $\sigma_{1}$ and $\sigma_{2}$, taking, for example, $T_{2}/T_{1}=\sqrt{2}$ in equation (5.2); the corresponding activation function is shown in the bottom plot of figure 11*a*, and the resulting long-term trajectory is displayed in figure 11*c*. Sharp turns at seemingly unpredictable angles due to the combination of the omega-turn mechanism and complex activation functions makes the trajectory strongly reminiscent of foraging behaviour [37].

## 6. Concluding remarks

In this study, we formulated a minimal model of neuromechanical undulatory locomotion of an elastic slender body in a dissipation-dominated environment using the RFT and coupled phase oscillators that form a CPG-like network. In contrast to existing CPG models, which assume an undulatory body motion, our network model preserves the motion reciprocity. Nonetheless, local phase modulation of each oscillator gives rise to robust undulatory locomotion in a self-organized manner, through mechanosensory feedback of the local mechanical load.

By numerical explorations, we obtained a stable, coherent undulatory motion in a broad parameter region and the emergent gait pattern reasonably agrees with various motions of *C. elegans* in different media. This process emerges from local symmetry breaking in the fore–aft resistive torque perceived by mechanoreceptors, which is then reflected at the network level by symmetry breaking with respect to the swimmer’s transverse plane, allowing non-reciprocal movement. This elementary mechanism thus explains the link between local and global dynamics at the origin of the motor activation wave, a property that could be extended to other sensory feedback, such as proprioception.

This coherent undulatory locomotion was further explored using Purcell’s three-link model. We found through theoretical and numerical analyses that a saddle-node bifurcation of periodic orbits occurs at a certain level of phase coupling. This indicates that the local CPG oscillators require a certain level of coupling to stabilize the network and that the sensory feedback cannot generate a stable gait on its own.

We then exploited this dynamical system structure to design manoeuvring strategies, by using the signal sensitivity as a control. Motivated by *C. elegans* behaviour on agar, we demonstrated through numerical explorations that our CPG model reproduces well the reverse motion and foraging behaviour known as the omega turn. Our deterministic model can thus include various predefined stereotyped behaviours, the so-called neuromechanical ‘templates’ [63] given by cycles and fixed points in the state space. Similarly, our model exhibits apparently random motion due to transient dynamics between these attractors. Recent data-driven modelling approaches may discover such attractors and the associated transients in the behavioural-state space [36,64,65].

One can also build upon this property to investigate, with a set of simple building blocks, complex long-term behaviours observed in some biological locomotors. Furthermore, this methodology of manipulating the nonlinear dynamical systems might offer a robust design method for redundant robot controllers [66].

In this study, we propose a general mechanism for gait adaptation through exteroceptive mechanosensory feedback, and remarkably, our numerical results are qualitatively in agreement with *C. elegans* behaviours, in particular on the decrease of wavelength of the emergent undulatory motion and amplification of internal actuation in high viscosity medium [52]. The current findings will be useful in modelling complex behavioural patterns and gait adaptations of animals.

The similar tendency of the changes in wavelength and internal actuation at the high viscosity regime is known in sperm motility [54,55]. In unicellular organisms with cilia and flagella, the undulatory motions are regulated by dynein motors, and mechanochemical oscillations of dynein actuation are reasonably represented by a coupled-oscillator system [67,68]. The exteroceptive feedback used in our manuscript could be used as a coarse-graining description of dynein regulation, although further studies should be warranted.

In our CPG model, the mechanosensation leads to a phase modulation that breaks the kinematic reversibility in the dissipative system. The non-reciprocity of the shape gait is recently found as the emergence of odd elasticity [43,69]. Such non-reciprocal material can generate effective work, and its mechanical efficiency merits further study.

In conclusion, our dynamical systems viewpoint is useful not only for understanding the extent of complexity and diversity found in animal locomotion but also for designing self-propelled, self-adaptive robots for a wide range of dissipative environments.

## Data Availability

The original code is freely available from the HAL online repository [70]. Supplementary material is available online [71].
